# Comparison of clinical outcomes of reperfusion therapies in in-hospital and community-onset acute ischemic stroke: A retrospective cohort study

**DOI:** 10.1097/MD.0000000000048777

**Published:** 2026-05-08

**Authors:** Cemile Haki, Tugce Yavas, Gulcin Koc Yamanyar, Kaya Sarac, Suat Kamisli

**Affiliations:** a Department of Neurology, University of Health Sciences, Bursa City Hospital, Bursa, Turkiye; b Department of Neurology, University of Health Sciences, Neurocritical Care Unit, Bursa City Hospital, Bursa, Turkiye; c Department of Radiology, University of Health Sciences, Bursa City Hospital, Bursa, Turkiye.

**Keywords:** acute ischemic stroke, in-hospital stroke, mechanical thrombectomy, recanalization therapies, treatment outcomes

## Abstract

In-hospital acute ischemic stroke (AIS) is a distinct clinical entity that may differ from community-onset AIS in terms of patient characteristics, treatment processes, and outcomes. This study aimed to compare the clinical features, treatment pathways, and functional outcomes of patients with in-hospital versus community-onset AIS who received recanalization therapies. This retrospective analysis included adult patients with AIS treated with intravenous tissue plasminogen activator and/or mechanical thrombectomy (MT) at a single center between July 2020 and January 2025. The patients were stratified based on whether stroke occurred during hospitalization or in the community. Patient demographics, treatment strategies, procedural time intervals, recanalization success, and clinical outcomes were assessed. The in-hospital stroke group had a higher prevalence of cardiac disease and malignancy. The patients in this group underwent groin puncture sooner after symptom onset, and all those who underwent MT achieved successful recanalization. Nevertheless, the rates of favorable functional recovery at 3 months (modified rankin scale ≤ 2) and in-hospital mortality were similar between the in-hospital and community-onset stroke groups. In-hospital stroke was not identified as a significant predictor of 3-month functional outcome in the multivariate analysis. Among patients who received recanalization therapies, in-hospital AIS cases (despite a higher comorbidity burden) can achieve clinical outcomes similar to those of community-onset strokes through early recognition and treatment initiation. These findings highlight the importance of structured in-hospital stroke protocols and the role of MT in in-hospital stroke management.

## 1. Introduction

Acute ischemic stroke (AIS) is among the most frequent neurological emergencies globally and is often accompanied by high mortality rate and persistent neurological deficits.^[1]^ The majority of clinically observed strokes are ischemic, resulting from cerebral circulation occlusion. Intravenous tissue plasminogen activator (IV t-PA) and/or mechanical thrombectomy (MT), used to achieve recanalization in AIS, have demonstrated efficacy in randomized clinical trials with high levels of evidence.^[2–4]^ Delays in the administration of these therapies result in greater neurological damage and worse clinical outcomes. This concept has been popularized by the slogan “time is brain.”^[5–7]^

Most AIS cases arise outside the hospital, with patients generally seeking evaluation in emergency departments following symptom onset.^[8]^ However, in some patients, stroke may occur during hospitalization due to underlying medical conditions, surgical procedures, or various interventional procedures, particularly those performed in cardiac or neurovascular fields.^[9–11]^

In patients with in-hospital stroke, the absence of the need to travel to the emergency department and immediate access to healthcare personnel may facilitate earlier symptom recognition and a shorter time to initiation of recanalization therapies. Despite these potential advantages, several studies have reported that patients with in-hospital stroke continue to experience diagnosis and management delay, often accompanied by poor functional outcomes.^[8,12–15]^ Collectively, these findings suggest that patients with in-hospital stroke constitute a unique population facing different challenges and patterns in diagnostic and treatment processes compared with those experiencing community-onset stroke.

This study aimed to evaluate the demographic, clinical, and procedural characteristics, as well as the functional outcomes, of patients with in-hospital AIS who received recanalization therapies and to compare these findings with those of community-onset stroke.

Furthermore, by identifying potential delays in decision-making and treatment in patients receiving recanalization therapy for in-hospitalstroke, this study seeks to support system-level improvements.

## 2. Materials and methods

This study included patients who were diagnosed with AIS and received recanalization therapies at Bursa City Hospital between July 2020 and January 2025. Our center is a large city hospital located in Bursa, Turkey, and has a fully equipped emergency department and a comprehensive stroke center operating 24/7.

Patients who were referred to our center for reperfusion therapy after experiencing a stroke at another hospital, those with incomplete follow-up data, those for whom 3-month functional assessment could not be performed, and patients aged < 18 years were excluded from the study.

Ethical approval was obtained from the Bursa City Hospital Clinical Research Ethics Committee (Approval date: April 16,2025; Decision No: 2025-8/6). Given the retrospective nature of the study, the requirement for individual informed consent was waived by the ethics committee. All procedures were conducted in accordance with the ethical principles of the Declaration of Helsinki.

In-hospital stroke was defined as the onset of new neurological symptoms compatible with AIS while a patient was hospitalized for a nonstroke illness or for a surgical or interventional procedure and subsequently received recanalization therapy. Community-onset stroke refers to AIS that developed prior to hospital admission and treated with recanalization therapy.

Baseline neurological deficit was quantified using the internationally validated National Institutes of Health Stroke Scale (NIHSS), with scores ranging from 0 to 42.^[16]^ The etiological classification of ischemic stroke was based on the Trial of ORG 10172 in Acute Stroke Treatment system.^[17]^

Patients who presented within 4.5 hours of symptom onset and had no contraindication to treatment received IV t-PA at a dose of 0.9 mg/kg. Treatment was initiated with a 10% bolus, with the remaining dose administered as an infusion over 1 hour.

The angiographic reperfusion status was graded according to the modified thrombolysis in cerebral infarction score, with grades 2b, 2c, and 3 considered to indicate successful reperfusion.^[2,18]^

Symptomatic intracranial hemorrhage (sICH) was defined according to the Heidelberg classification as the presence of new or enlarged intracranial hemorrhage on imaging alongside an increase of ≥ 4 points in the NIHSS score or any neurological deterioration attributable to the hemorrhage.^[19]^

The patients’ functional outcomes 3-month poststroke were determined using the modified rankin scale (mRS), with scores 0 to 2 indicating a “good functional outcome” and scores 3 to 6 indicating a “poor functional outcome.”

Patients with community-onset stroke treated with reperfusion therapies and those with in-hospital stroke were compared in terms of demographic characteristics, stroke risk factors, stroke characteristics (admission NIHSS score, Alberta stroke program early computed tomography score [ASPECTS], stroke location and etiology, wake-up stroke), treatment interventions (IV t-PA, MT, IV t-PA + MT), time intervals (symptom-to-IV t-PA time, symptom-to-groin puncture time), sICH, reperfusion success, and functional outcomes.

In addition, the clinical ward where the patient was admitted, primary medical condition at the time of admission, and any history of operations or procedures were recorded. The independent association of in-hospital AIS with 3-month functional status (good functional outcome, measured by mRS) in patients treated with reperfusion therapies was evaluated via multivariate logistic regression analysis.

In MT procedures, in accordance with current clinical guidelines, stent-retriever devices, aspiration-based approaches, balloon angioplasty, or combinations of these techniques were employed as appropriate. In-hospital stroke patients were evaluated for MT candidacy based on imaging-confirmed large vessel occlusion and a premorbid mRS score of ≤ 2, in accordance with current international guidelines. Given the high comorbidity burden of this population, treatment decisions were made on an individualized basis through multidisciplinary team assessment, with explicit consideration of recent surgical history, active malignancy, anticoagulation status, and overall clinical stability. Recent surgery was not regarded as an absolute contraindication to MT; eligibility was determined after careful evaluation of procedural bleeding risk and hemodynamic status. In patients receiving anticoagulant therapy, decision-making followed guideline-based safety criteria. MT was performed when the anticipated clinical benefit was judged to outweigh the procedural and hemorrhagic risks.

### 2.1. Statistical analysis

All statistical analyses were conducted using the IBM SPSS software package for Windows (version 27.0; IBM Corp.). Statistical inference was based on 2-sided testing, with a significance threshold set at *P* < .05. Distributional patterns of continuous variables were evaluated through graphical examination, including histogram and normal probability (Q–Q) plots, supplemented by formal normality testing using the Shapiro-Wilk test. Given the relatively small sample size of the in-hospital stroke group (n = 31), the Shapiro-Wilk test was considered the most appropriate formal assessment. Variables meeting normality assumptions were analyzed using parametric tests, while those deviating from normality were analyzed using nonparametric equivalents. Data conforming to a normal distribution were summarized using mean values with corresponding standard deviations. Skewed variables were described using median values and interquartile ranges. Discrete variables were expressed as absolute numbers and percentages.

Comparative analyses of continuous variables were conducted using parametric or nonparametric tests (Student *t*-test or Mann-Whitney *U* test), selected according to distribution characteristics. Relationships between categorical variables were examined using the chi-squared or exact test, including Fisher exact and Fisher–Freeman–Halton tests, when applicable. To explore the independent determinants of favorable functional status at 3 months, logistic regression modeling was employed. Subsequently, candidate variables identified as significant in preliminary univariate models were incorporated into the final multivariable regression analysis.

## 3. Results

The study population comprised 472 patients with AIS, including 441 with community-onset stroke and 31 with in-hospital stroke.Their mean age was 69.31 ± 12.27 years. Patients in the in-hospital stroke group were younger (*P = *.024) and had a higher incidence of cardiac disease (*P = *.015) and malignancy (*P* = .024). No statistically significant differences were observed between the groups in terms of sex, hypertension, diabetes mellitus (DM), dyslipidemia, prior stroke, prior transient ischemic attack, smoking, or alcohol use (Table 1).

**Table 1:** Baseline patient profile and comorbid conditions in community-onset and in-hospital stroke.

	All patients (n = 472)	Stroke		*P*
		Community onset (n = 441)	In-hospital (n = 31)	
Age, yrs	69.31 ± 12.27	69.65 ± 12.17	64.52 ± 12.79	**.024** *
Sex				
Male	236 (50.00)	218 (49.43)	18 (58.06)	.457†
Female	236 (50.00)	223 (50.57)	13 (41.94)	
HT	317 (67.16)	298 (67.57)	19 (61.29)	.602†
DM	158 (33.47)	146 (33.11)	12 (38.71)	.658†
Heart disease	259 (54.87)	235 (53.29)	24 (77.42)	**.015** †
Dyslipidemia	118 (25.00)	108 (24.49)	10 (32.26)	.453†
Previous stroke history	83 (17.58)	79 (17.91)	4 (12.90)	.642†
Prior TIA history	24 (5.08)	24 (5.44)	0 (0.00)	.391‡
Malignancy	18 (3.81)	14 (3.17)	4 (12.90)	**.024** ‡
Smoking	98 (20.76)	87 (19.73)	11 (35.48)	.063†
Alcohol use	20 (4.24)	20 (4.54)	0 (0.00)	.633‡

Normally distributed continuous variables are expressed as mean ± SD. Categorical variables are expressed as frequency (percentage).

DM = diabetes mellitus, HT = hypertension, n = number of patients, TIA = transient ischemic attack.

* Student’s *t*-test.

† chi-squared test.

‡ Fisher exact test. Statistical significance is indicated by bold text.

The admission NIHSS scores (*P = *.008) and ASPECTS values (*P = *.031) were higher in the in-hospital stroke group. At 3 months, functional independence (mRS 0–2) was achieved by 211 community-onset stroke patients (47.85%) and 13 in-hospital stroke patients (41.94%). Comparison of the 2 groups revealed similar distributions regarding stroke location and etiology, wake-up stroke status, pretreatment antiplatelet and anticoagulant use, sICH, in-hospital death, discharge mRS, as well as functional and mortality outcomes at 3 months (Table 2).

**Table 2:** Clinical and stroke characteristics.

	All patients (n = 472)	Stroke		*P*
		Community onset (n = 441)	In-hospital (n = 31)	
Stroke location				
Posterior	67 (14.19)	63 (14.29)	4 (12.90)	1.000¶
Anterior	402 (85.17)	375 (85.03)	27 (87.10)	
Both	3 (0.64)	3 (0.68)	0 (0.00)	
Etiology				
LAA	101 (21.40)	97 (22.00)	4 (12.90)	.777¶
CE	172 (36.44)	159 (36.05)	13 (41.94)	
SVO	20 (4.24)	19 (4.31)	1 (3.23)	
ODC	15 (3.18)	14 (3.17)	1 (3.23)	
UND	164 (34.75)	152 (34.47)	12 (38.71)	
Wake-up stroke	58 (12.29)	55 (12.47)	3 (9.68)	1.000‡
Pretreatment antiplatelet use	203 (43.01)	185 (41.95)	18 (58.06)	.118†
Pretreatment anticoagulant use	83 (17.58)	76 (17.23)	7 (22.58)	.609†
Admission NIHSS score	11.62 ± 5.97	11.43 ± 5.78	14.39 ± 7.84	**.008** *
ASPECTS				
≤ 9	93 (19.70)	92 (20.86)	1 (3.23)	**.031** †
10	379 (80.30)	349 (79.14)	30 (96.77)	
sICH	45 (9.53)	43 (9.75)	2 (6.45)	.756‡
In-hospital mortality	83 (17.58)	75 (17.01)	8 (25.81)	.317†
Discharge mRS score	3 (1–5)	3 (1–5)	4 (2–6)	.390§
3-Month mRS score	3 (1–5)	3 (1–5)	3 (1–6)	.305§
3-Month good functional outcome (mRS 0–2)	224 (47.46)	211 (47.85)	13 (41.94)	.652†
3-Month mortality	99 (20.97)	91 (20.63)	8 (25.81)	.649†

Descriptive statistics for continuous variables were selected based on distribution, with mean ± SD applied to normally distributed data and median values to nonnormal data (1st quartile–3rd quartile). Categorical variables are expressed as frequency (percentage).

ASPECTS = Alberta stroke program early computed tomography score, CE = cardioembolism, LAA = large artery atherosclerosis, mRS = modified Rankin Scale, n = number of patients, NIHSS = National Institutes of Health Stroke Scale, ODC, = other determined cause, sICH = symptomatic intracranial hemorrhage, SVO = small vessel occlusion, UND = undetermined etiology.

* Student’s *t*-test.

† chi-squared test.

‡ Fisher exact test; Fisher–Freeman–Halton test.

§ Mann–Whitney *U* test. Statistical significance is indicated by bold text.

The symptom-to-groin puncture time was shorter in the in-hospital stroke group (*P = *.003). Among patients with community-onset stroke, MT led to successful reperfusion in 246 of 275 treated cases (89.45%). In comparison, successful reperfusion was obtained in every patient treated with MT in the in-hospital stroke group (n = 22). Comparative analysis revealed similar treatment modality, symptom-to-IV t-PA time, or overall reperfusion success rates in both groups (Table 3).

**Table 3:** Treatment times and recanalization therapies.

	All patients (n = 472)	Stroke		*P*
		Community onset (n = 441)	In-hospital (n = 31)	
Treatment				
IV t-PA	175 (37.08)	166 (37.64)	9 (29.03)	.358†
IV t-PA + MT	66 (13.98)	63 (14.29)	3 (9.68)	
MT	231 (48.94)	212 (48.07)	19 (61.29)	
Symptom-to-IV t-PA time, min.	150 (110–208)	150 (110–208)	135 (60–200)	.215§
Symptom-to-groin puncture time, min.	189 (135–270)	195 (135–280)	124 (100–165)	**.003** §
Symptom-to-treatment time, min.	173.5 (120–240)	180 (120–240)	135 (60–215)	**.014** §
Reperfusion success (mTICI)				
0	14 (4.71)	14 (5.09)	0 (0.00)	.847¶
1	12 (4.04)	12 (4.36)	0 (0.00)	
2a	3 (1.01)	3 (1.09)	0 (0.00)	
2b	47 (15.82)	44 (16.00)	3 (13.64)	
2c	9 (3.03)	9 (3.27)	0 (0.00)	
3	212 (71.38)	193 (70.18)	19 (86.36)	
Reperfusion success (mTICI)				
Successful reperfusion (mTICI 2b–3)	268 (90.24)	246 (89.45)	22 (100.00)	.145‡
Failed reperfusion (mTICI < 2b)	29 (9.76)	29 (10.55)	0 (0.00)	

Non-normally distributed continuous variables are expressed as median (1st quartile–3rd quartile). Categorical variables are expressed as frequency (percentage).

IV t-PA = intravenous tissue plasminogen activator, MT = mechanical thrombectomy, mTICI = modified thrombolysis in cerebral infarction score, n = number of patients.

† chi-squared test.

‡ Fisher exact test; Fisher–Freeman–Halton test.

§ Mann–Whitney *U* test. Statistical significance is indicated by bold text.

**Figure 1.**
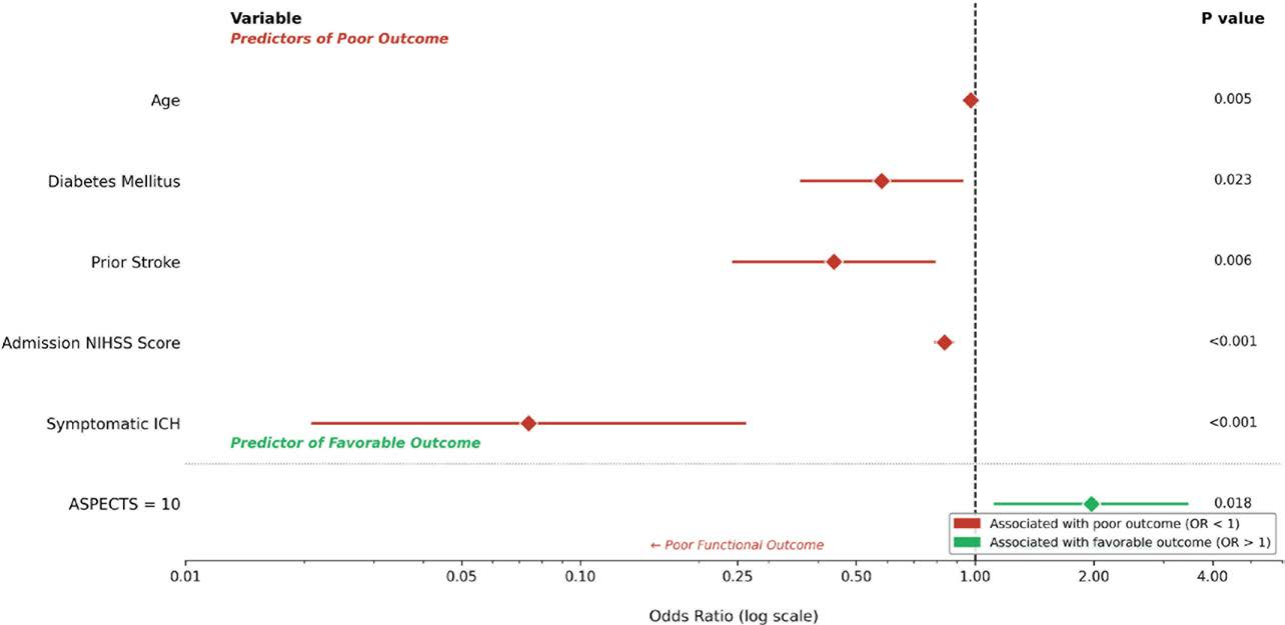
Forest plot illustrating the adjusted ORs with 95% CIs for independent predictors of favorable 3-month functional outcome (mRS 0–2) identified in the multivariable logistic regression analysis. Variables with OR < 1 are independently associated with poor functional outcome; ASPECTS = 10 (OR > 1) is independently associated with favorable outcome. ASPECTS = Alberta stroke program early computed tomography score, CI = confidence interval, DM = diabetes mellitus, mRS = modified rankin scale, NIHSS = National Institutes of Health Stroke Scale, OR = odds ratio, sICH = symptomatic intracranial hemorrhage,.

Multivariable logistic regression analysis revealed that older age (odds ratio [OR]: 0.975; 95% confidence interval [CI]: 0.957–0.992; *P = *.005), DM (OR: 0.580; 95% CI: 0.363–0.927; *P = *.023), prior stroke history (OR: 0.439; 95% CI: 0.244–0.788; *P = *.006), higher admission NIHSS scores (OR: 0.836; 95% CI: 0.795–0.878; *P* < .001), and sICH (OR: 0.074; 95% CI: 0.021–0.261; *P* < .001) were independently associated with the absence of a good functional outcome at 3 months. Logistic regression analysis revealed that an ASPECTS score of 10 independently predicted a favorable functional outcome at 3 months (OR: 1.970; 95% CI: 1.126–3.448; *P = *.018; Table 4). These findings are further illustrated in Figure 1.

**Table 4:** Univariate and multivariate predictors of 3-month functional outcomes.

	Univariate		Multivariate	
	OR (95% CI)	*P*	OR (95% CI)	*P*
Age, yrs	0.972 (0.957–0.987)	**< .001**	0.975 (0.957–0.992)	**.005**
Sex, female	0.844 (0.588–1.211)	.357		
HT, yes	0.751 (0.511–1.103)	.145		
DM, yes	0.630 (0.428–0.929)	**.02**	0.580 (0.363–0.927)	**.023**
Heart disease, yes	0.845 (0.587–1.215)	.363		
Dyslipidemia, yes	1.046 (0.690–1.588)	.831		
Prior stroke history, yes	0.470 (0.285–0.775)	**.003**	0.439 (0.244–0.788)	**.006**
Prior TIA history, yes	0.934 (0.409–2.128)	.87		
Malignancy, yes	2.283 (0.842–6.188)	.105		
Smoking, yes	1.328 (0.850–2.073)	.213		
Alcohol use, yes	2.121 (0.831–5.415)	.116		
Stroke location, anterior	0.920 (0.548–1.542)	.751		
Etiology				
LAA	0.739 (0.474–1.154)	.183		
CE	0.977 (0.671–1.423)	.904		
SVO	2.689 (1.015–7.122)	**.047**	1.804 (0.566–5.755)	.319
ODC	1.275 (0.455–3.575)	.644		
UND	1.045 (0.715–1.527)	.821		
Wake-up stroke, yes	0.818 (0.470–1.425)	.479		
Pretreatment antiplatelet use, yes	0.860 (0.597–1.240)	.419		
Pretreatment anticoagulant use, yes	0.869 (0.540–1.399)	.563		
Admission NIHSS score	0.837 (0.802–0.872)	**< .001**	0.836 (0.795–0.878)	**< .001**
ASPECTS = 10	2.075 (1.289–3.340)	**.003**	1.970 (1.126–3.448)	**.018**
sICH, yes	0.067 (0.020–0.218)	**< .001**	0.074 (0.021–0.261)	**< .001**
Treatment				
IV t-PA	2.330 (1.590–3.415)	**< .001**	0.689 (0.343–1.382)	.294
IV t-PA + MT	0.977 (0.580–1.646)	.932		
MT	0.459 (0.317–0.663)	**< .001**	0.857 (0.450–1.632)	.639
Symptom-to-treatment time, min.	0.999 (0.997–1.000)	.125		
Stroke type, in-hospital	0.787 (0.377–1.646)	.525		
Nagelkerke R^2^	-		0.365	

ASPECTS = Alberta stroke program early computed tomography score, CE = cardioembolism, CI = confidence interval, DM = diabetes mellitus, HT = hypertension, IV t-PA = intravenous tissue plasminogen activator, LAA = large artery atherosclerosis, MT = mechanical thrombectomy, NIHSS = National Institutes of Health Stroke Scale, ODC = other determined cause, OR = odds ratio, sICH = symptomatic intracranial hemorrhage, SVO = small vessel occlusion, TIA = transient ischemic attack, UND = undetermined etiology.

Table 5 summarizes the demographic characteristics, reasons for admission, comorbidities, antithrombotic treatment status, recanalization methods, and clinical outcomes of patients with in-hospital stroke.

**Table 5:** Detailed profile of in-hospital stroke patients receiving recanalization therapy.

Patient	Age	Sex	Reason for Admission	Ward/Unit	Comorbidities	Time of Stroke (d)	Operation/Invasive Procedure	Antiplatelet/Antithrombotic at Admission	IV t-PA: 1 — IV t-PA + MT: 2 — MT: 3	IV t-PA Contraindication	Recanalization Success (TICI)	Cause of In-Hospital Mortality
1	82	Female	Acute coronary syndrome	Coronary ICU	-	8	No	Antiplatelet	1	IV t-PA	IV t-PA	No
2	48	Female	Infective endocarditis	Cardiology Ward	Mechanical MVR; COVID-19 pneumonia	17	No	VKA	3	VKA use (INR > 1.7)	TICI 2B	Septic shock
3	81	Male	COVID-19 pneumonia	Pulmonary ICU	CAD	15	No	LMWH	1	IV t-PA	IV t-PA	ARDS; septic shock
4	42	Female	Carpal tunnel syndrome, elective surgery	Hand Surgery Ward	-	3	No	No	1	IV t-PA	IV t-PA	No
5	76	Male	Dilated cardiomyopathy; ACS	Cardiology Ward	Ischemic stroke; COPD	4	Yes	LMWH; antiplatelet	3	LMWH at therapeutic dose	TICI 3	No
6	68	Male	BPH; prostatectomy	Urology Ward	-	1	Yes	No	3	Major surgery	TICI 3	No
7	77	Male	Medullary sponge kidney; urinary tract infection	Nephrology Ward	-	17	No	Antiplatelet	1	IV t-PA	IV t-PA	Multiple-organ failure
8	79	Female	Atrioventricular block	Coronary ICU	CAD; AF	8	Yes	Antiplatelet	3	Major surgery	TICI 3	No
9	64	Male	Inferior myocardial infarction	Coronary ICU	CABG	4	No	LMWH; antiplatelet	3	LMWH at therapeutic dose	TICI 3	No
10	55	Female	Type 1 aortic dissection	CVS Ward	-	1	Yes	LMWH	3	Major surgery; aortic dissection; thrombocytopenia	TICI 3	Ventricular fibrillation
11	71	Male	Subacute inferior myocardial infarction	Cardiology Ward	Total hip replacement	16	Yes	LMWH	3	LMWH at therapeutic dose	TICI 3	Septic shock
12	57	Male	Pulmonary thromboembolism	Emergency Department	-	1	No	No	1	IV t-PA	IV t-PA	No
13	43	Female	Intestinal obstruction; colectomy	General Surgery ICU	Colon cancer	1	Yes	LMWH	3	Major surgery; LMWH at therapeutic dose	TICI 2B	Multiple-organ failure
14	66	Male	Gluteal thermal burn	Burn Unit	CABG; Parkinson disease	2	No	LMWH	3	LMWH at therapeutic dose	TICI 3	No
15	55	Female	Lung cancer; nasopharyngeal cancer	Pulmonary Ward	Hypothyroidism	1	No	No	3	Large infarct (total MCA infarct)	TICI 3	No
16	60	Male	Atrial flutter ablation	Cardiology Ward	COPD	1	No	DOAC	3	DOAC use	TICI 3	ARDS; AV block
17	78	Male	CAD	Coronary ICU	AF	1	No	LMWH	3	LMWH at therapeutic dose	TICI 3	No
18	48	Male	Inferior myocardial infarction	Cardiology Ward	BPH	1	No	LMWH; antiplatelet	3	LMWH at therapeutic dose	TICI 2B	No
19	54	Male	Carotid endarterectomy	CVS Ward	Ischemic stroke	2	Yes	Antiplatelet	3	Major surgery	TICI 3	No
20	55	Male	NSTEMI	Coronary ICU	Ischemic stroke; CABG	2	No	LMWH	1	IV t-PA	IV t-PA	No
21	67	Female	Gastroenteritis	Emergency Department	-	1	No	No	2	IV t-PA	TICI 3	No
22	82	Female	ACS; ventricular tachycardia	Coronary ICU	-	1	Yes	Antiplatelet; heparin	3	LMWH at therapeutic dose	TICI 3	Subarachnoid hemorrhage
23	55	Male	Lumbar disc herniation	Emergency Department	CAD	1	No	Antiplatelet	2	IV t-PA	TICI 3	No
24	87	Female	Meningitis	Infectious Disease Ward	ICD	5	No	Antiplatelet	3	Wake-up stroke	TICI 3	No
25	58	Male	NSTEMI	Coronary ICU	CABG	1	No	LMWH; antiplatelet	2	IV t-PA	TICI 3	No
26	67	Male	Stable angina pectoris	Coronary ICU	-	1	No	No	1	IV t-PA	IV t-PA	No
27	43	Female	Ovarian mass (benign endometrioma)	Gynecology Ward	Mechanical MVR	2	Yes	LMWH	3	Major surgery and LMWH at therapeutic dose	TICI 3	No
28	64	Female	Decompensated CHF; pneumonia	Cardiology Ward	Mechanical AVR	13	No	LMWH; VKA	3	LMWH at therapeutic dose; VKA use	TICI 3	No
29	72	Male	CAD	Coronary ICU	CAD	1	No	LMWH; antiplatelet	1	IV t-PA	IV t-PA	No
30	72	Male	Lung cancer; pulmonary thromboembolism	Pulmonary Ward	COPD; BPH	5	No	LMWH	3	LMWH at therapeutic dose	TICI 3	No
31	74	Female	Pneumonia	Pulmonary Ward	Breast cancer	5	No	No	1	IV t-PA	IV t-PA	No

ACS = acute coronary syndrome, AF = atrial fibrillation, ARDS = acute respiratory distress syndrome, AV = atrioventricular, AVR = aortic valve replacement, BPH = benign prostatic hyperplasia, CABG = coronary artery bypass grafting, CAD = coronary artery disease, CHF = congestive heart failure, COPD = chronic obstructive pulmonary disease, CVS = cardiovascular surgery, DOAC = direct oral anticoagulant, ICD = implantable cardioverter-defibrillator, ICU = intensive care unit, INR = international normalized ratio; COVID-19, coronavirus disease 2019, IV t-PA = intravenous tissue plasminogen activator, LMWH = low molecular weight heparin, MCA = middle cerebral artery, MT = mechanical thrombectomy, mTICI = modified thrombolysis in cerebral infarction score, MVR = mitral valve replacement, NSTEMI = non-ST-elevation myocardial infarction, VKA = vitamin K antagonist.

## 4. Discussion

This study compared community-onset stroke and in-hospital stroke in terms of clinical outcomes and treatment characteristics. At the 3-month follow-up, the functional outcome distributions and mortality did not significantly differ between the groups. Nevertheless, patients with in-hospital stroke had a higher prevalence of cardiac disease and malignancy, and nearly one-third experienced stroke in the postoperative period or following an interventional procedure. In addition, patients with in-hospital stroke underwent endovascular treatment with shorter delays after symptom recognition and demonstrated high reperfusion rates.

In patients who develop in-hospital stroke, factors such as advanced age, greater comorbidity burden, critical illnesses requiring hospitalization, delays in symptom recognition, and disruptions in diagnostic-treatment workflows potentially contribute to less-favorable prognoses than those observed in patients with community-onset stroke.^[8,12,20–22]^

Previous studies reporting on patients with in-hospital AIS treated with reperfusion therapies have generally demonstrated higher mortality and morbidity rates, along with a markedly prolonged length of hospitalization compared with patients with community-onset stroke.^[23–26]^ Nevertheless, several studies with relatively small case series treated with MT have reported comparable mortality and morbidity rates between patients with community-onset and in-hospital stroke.^[27]^ In our cohort, we observed that both groups achieved comparable functional recovery and survival at the 3-month follow-up.

Hospitalized patients represent a high-risk population for acute stroke. The major contributors to increased in-hospital stroke risk include severe comorbid conditions (cardiac disease, DM, hypertension, cardiovascular disease, malignancy), surgeries and invasive procedures, acute or serious illnesses requiring hospitalization, perioperative interruption of antiplatelet or anticoagulant therapy, immobility, and coagulation disorders.^[9,28–31]^ Analysis of our study population showed that the in-hospital stroke group included a higher proportion of patients with underlying cardiac disease (*P = *.015) and malignancy (*P = *.024) than the community-onset stroke group, consistent with previously published data.^[24,32,33]^

The literature generally reports that patients who develop in-hospital stroke are older than those who develop community-onset stroke.^[21]^ Contrarily, several investigations focusing on patients treated with recanalization therapies have found no statistically meaningful age differences, or comparable mean ages, between the in-hospital and community-onset stroke groups.^[32–34]^ In our study, consistent with the findings of Yoo et al,^[24]^ patients in the in-hospital stroke group who received recanalization therapy were younger on average. This finding may be related to our center’s patient profile and admission criteria as well as the selection of patients for recanalization who were more hemodynamically stable and had fewer severe comorbidities.

In our study, the baseline NIHSS scores were significantly higher among individuals with in-hospital stroke than among those in the community-onset stroke group. This finding is consistent with earlier reports indicating that strokes occurring during hospitalization are commonly associated with more pronounced neurological impairment.^[8,33,35]^ Furthermore, the ASPECTS values were higher in the in-hospital than in the community-onset stroke group. Wang et al^[9]^ evaluated patients with perioperative ischemic stroke and reported significantly higher ASPECTS than in patients with community-onset stroke.

On non-neurology wards, relatively lower stroke awareness among healthcare personnel and the presence of conditions such as intubation, postoperative sedation, or altered consciousness may obscure stroke signs and delay recognition, resulting in delayed initiation of reperfusion therapies.^[15,29]^ Previous studies have reported variable results regarding symptom-to-groin puncture time in in-hospital versus community-onset stroke. Akbik et al, in their comprehensive analysis of published data, reported a mean diagnosis-to-groin puncture time of 165 minutes for patients with in-hospital stroke.^[23]^ Whereas longer delays have been described in some cohorts,^[13]^ our study demonstrated a shorter median interval of 124 minutes in hospitalized stroke cases compared with community-onset stroke cases (*P = *.003).

Although the rates of cardiac disease and malignancy were significantly higher in the in-hospital stroke group in our cohort, no significant differences was observed between the in-hospital and community-onset stroke groups in terms of 3-month functional outcomes and mortality. The possible explanations for this observation are as follows: First, the considerably shorter symptom-to-groin puncture time observed in in-hospital stroke cases may have conferred a time advantage that partially offset differences in clinical outcomes. Second, the high level of institutional experience with MT at our center and the high rate of successful recanalization in in-hospital stroke patients may have improved the outcomes in this group. Third, the higher ASPECTS values observed among patients with in-hospital stroke could reflect better preserved cerebral tissue, offering an additional advantage that favorably influenced functional outcomes. Furthermore, the limited sample size of the in-hospital stroke group may have prevented some true differences from reaching statistical significance. Indeed, the absence of an independent association between in-hospital stroke status and good 3-month functional outcome in multivariate analysis indicates that the principal determinants of clinical course in this patient subgroup are the following variables: age, admission NIHSS score, DM, prior stroke, and sICH.

It has been reported that patients who develop AIS in the hospital are less likely to receive IV t-PA due to bleeding risk related to recent surgery or procedures, uncertainty regarding the exact time of symptom onset, and other contraindications.^[30,36]^ In the present study, the most frequent contraindication among patients who could not receive IV t-PA was anticoagulant use (n = 13), followed by recent surgery and clinical conditions associated with a high risk of hemorrhage (total n = 6). Less common outcomes included wake-up stroke (n = 1) and extensive infarct territory (n = 1). These findings suggest that anticoagulant exposure, perioperative status, and clinical scenarios conferring high hemorrhagic risk considerably limit IV t-PA eligibility in in-hospital AIS. Although IV t-PA was less frequently administeredin the in-hospital stroke group than in the community-onset stroke group (29.0% vs 37.6%), this difference did not reach statistical significance. MT utilization and recanalization outcomes were comparable across the 2 groups. Notably, the rate of successful reperfusion (modified thrombolysis in cerebral infarction score 2b–3) in the in-hospital stroke group was 100%. Our findings are consistent with the results of the review and meta-analysis conducted by Amoukhteh et al, which reported high successful recanalization rates in patients with in-hospital stroke.^[37]^ Despite restricted access to IV t-PA in in-hospital cases due to clinical constraints, the high recanalization success observed suggests that endovascular strategies, such as MT, may represent an important alternative treatment approach for appropriately selected patients in this population.

The literature suggests that strokes may occur in hospitalized patients during the perioperative period, particularly following cardiac procedures and other surgical or interventional interventions.^[38,39]^ In our cohort, stroke occurred during or after an operation or invasive procedure in 29% of in-hospital stroke cases. This finding underscores the vulnerability of hospitalized patients to stroke owing to their underlying systemic disease burden and the medical interventions they undergo, emphasizing the critical importance of preventive strategies in this subgroup. In the present study, in-hospital stroke cases were most commonly monitored on cardiology (48.4%), cardiovascular surgery (6.5%), and non-cardiac intensive care (6.5%) wards. In a study by Matsubara et al,^[25]^ 6 (60%) of 10 patients who underwent MT for in-hospital large vessel occlusion were followed up in the cardiology ward, whereas the remainder were followed up in the hematology, otolaryngology, urology, and gastroenterology wards.

Cardioembolism has been reported as a common etiologic cause of in-hospital stroke, particularly among patients undergoing MT.^[25,26,32,40]^ In the present study, cardioembolic causes comprised the largest etiologic subgroup among in-hospital stroke cases (41.9%). This finding supports the concept that patients with cardiac comorbidities are at a particularly high risk.

It is important to implement stroke-recognition and early-management training for nurses and other healthcare personnel working on wards that frequently care for patients at an increased risk for in-hospital stroke, such as cardiology, surgical, and intensive care units. Moreover, to reduce postoperative stroke risk, perioperative interruption and reinitiation of antiplatelet or anticoagulant therapies should be appropriately managed, perioperative hypotension should be avoided, and anesthesia should be carefully selected.^[30]^

To avoid missing the therapeutic time window in hospitalized patients with AIS, rapid stroke-recognition, emergent neuroimaging, immediate contact with neurology and the interventional team, timely identification of patients likely to benefit from recanalization therapies, and implementation of in-hospital stroke protocols to reduce potential treatment delays are essential.^[29]^

## 5. Conclusion

This study reflects the experience of a single tertiary care institution and is based on retrospectively collected data. Therefore, the patient population may be more complex than that of other settings. Because of the retrospective study design, the time-point data for each step of the workflow were not completely recorded for all patients. This lack of detailed time metrics prevented us from performing the comprehensive process comparisons frequently emphasized in the literature and limited our ability to evaluate time performance specifically in the in-hospital stroke subgroup. Furthermore, the substantial difference in group sizes (with only 31 in-hospital stroke patients compared with 441 community-onset cases) and the retrospective nature of the study precluded the application of propensity score matching or other bias-adjustment methods that would ideally be prespecified in the study design. Residual confounding due to baseline differences between groups therefore cannot be fully excluded.

In Turkey, studies investigating recanalization and outcomes specifically in in-hospital stroke cases are scarce. This relative scarcity of data highlights the validity of our work.

In summary, our results indicate that in-hospital stroke represents a complex, multifactorial problem: despite the high comorbidity burden, early recognition and rapid intervention (shorter symptom-to-treatment times) can yield 3-month functional outcomes comparable to those of community-onset strokes. Strengthening diagnostic–treatment pathways and increasing awareness among healthcare personnel can further improve clinical outcomes in this high-risk group.

## Acknowledgments

The authors received no financial support for the research, authorship, or publication of this article. The authors declare no conflicts of interest.

## Author contributions

**Conceptualization:** Cemile Haki, Suat Kamisli.

**Data curation:** Tugce Yavas.

**Formal analysis:** Cemile Haki, Suat Kamisli.

**Investigation:** Cemile Haki, Tugce Yavas, Gulcin Koc Yamanyar, Kaya Sarac.

**Methodology:** Cemile Haki, Suat Kamisli.

**Project administration:** Cemile Haki, Suat Kamisli.

**Resources:** Gulcin Koc Yamanyar, Kaya Sarac.

**Supervision:** Suat Kamisli.

**Validation:** Cemile Haki, Tugce Yavas, Suat Kamisli, Gulcin Koc Yamanyar, Kaya Sarac.

**Visualization:** Cemile Haki.

**Writing – original draft:** Cemile Haki, Tugce Yavas.

**Writing – review & editing:** Cemile Haki, Tugce Yavas, Gulcin Koc Yamanyar, Kaya Sarac, Suat Kamisli.
